# Design of Facial Recognition System Based on Visual Communication Effect

**DOI:** 10.1155/2021/1539596

**Published:** 2021-12-09

**Authors:** Xuhui Fu

**Affiliations:** Culture and Art Management of Hunan University, Korea, Guangzhou City 62399, Republic of Korea

## Abstract

At present, facial recognition technology is a very cutting-edge science and technology, and it has now become a very hot research branch. In this research, first, the thesis first summarized the research status of facial recognition technology and related technologies based on visual communication and then used the OpenCV open source vision library based on the design of the system architecture and the installed system hardware conditions. The face detection program and the image matching program are realized, and the complete face recognition system based on OpenCV is realized. The experimental results show that the hardware system built by the software can realize the image capture and online recognition. The applied objects are testers. In general, the OpenCV-based face recognition system for testers can reliably, stably, and quickly realize face detection and recognition in this situation. Facial recognition works well.

## 1. Preface

In the context of continuous advancement in technology, traditional identification methods continue to withstand more and more challenges, and the accuracy of identification can no longer meet the needs. Nowadays, the recognition of biometric information is gradually attracting attention.

As one of the biometric recognition technologies, facial recognition technology occupies a pivotal position in identity recognition. [1] Facial recognition, as a multidisciplinary cross-integration technology, mainly includes technologies such as computers, information processing, and image feature recognition. Facial recognition technology has made great progress in recent years. Nature contains a variety of information, such as sound information, electric field information, visual information, magnetic field information, and thermal field information. Among them, visual information contains a huge amount of data [2]. How to extract useful information from these data? And applying these data to change our actual life and work will be of great significance. The proposition of “how to distinguish human identities” is embodied in the field of image processing technology, which is to use image processing methods to use specific algorithms to extract in-depth characteristic information related to individuals and to use the obtained characteristic information to distinguish other acquired characteristic information [3]. Therefore, according to the uniqueness of the characteristic information identification, the identification and confirmation of different people can be realized, so as to further use this as a credential to realize a technology for supervision, management, and control of individuals.

According to the development process of facial recognition technology, it can be divided into the following three recognition methods. (1) The facial recognition method is based on geometric features. The method is simple, but the recognition accuracy is low, and the recognition effect is not ideal, but it provides a new research idea for face recognition [4]. (2) The face recognition method based on template matching is implemented based on the global features of the face to be recognized. It is implemented based on the global features of the face to be recognized. First, the facial image is normalized and normalized. After morphological processing such as scale normalization, histogram equalization, and corrosion expansion, an 8*∗*8 template is then used to extract features from it using a method similar to the LBP algorithm to obtain a 64-bit hash code. There are *n* images in the training set. We will process all these *n* images by the above method to obtain the hash codes of *n* training sets. Similarly, we will process a face image to be recognized in the same way. Finally, we will wait. The hash code that recognizes the face image is compared with the hash code of each person in the training set. The main purpose is to compare the Hamming distance between them. The hash code of the face image to be recognized is compared with the hash code of each image in the training set. After hash code comparison, *n* Hamming distances are generated, the minimum value of these *n* Hamming distances is calculated, and it is determined that the human face image to be recognized is the most similar to the picture in the training set that produces the distance [5]. (3) The model-based facial recognition method, through the method of sample analysis, to obtain the template is used in the model; common models such as the human face recognition method are based on the hidden Markov model [6]; the facial image is regarded as the different expressions of the various organs of the face The face organ is regarded as an abstract state; the state and the form of expression are related through two random processes; a large number of face pictures are used as the actual training set, the model is trained, and this is determined by a certain algorithm. The optimal parameters of the model so that every person in the training set have an optimal model to match with it. In face recognition, calculate which model has the highest matching degree with the face feature to be recognized; then, the person to be identified and the person corresponding to the model in the training set are grouped into one category, that is, to find out which model is the most likely to produce the expression [7].

**Figure 1:**
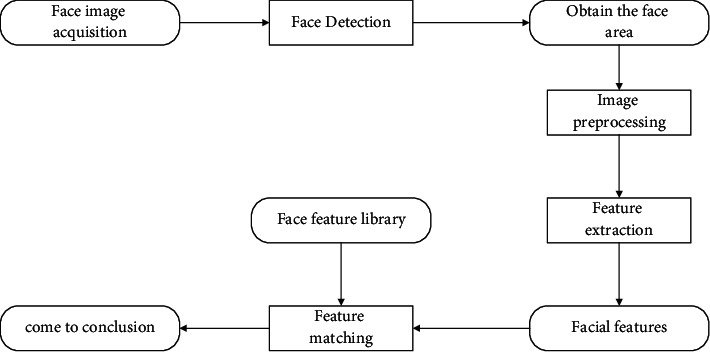
Schematic diagram of facial recognition.

Facial recognition is the process of comparing the images collected by the camera with the facial images in the database, using relevant computer algorithms to analyze and extract effective identification feature information [8–10]. It combines multiple research fields such as image processing, computer graphics, visualization technology, human physiology, and pattern recognition. The basic process of facial recognition is shown in Figure 1, and its technical process is divided into the following main parts.

Aiming at the deficiencies in the current commonly used facial recognition technologies, this topic relies on Linux as the development platform, uses the Qt5 development environment, and uses the relevant image processing algorithms of the computer image processing software OpenCV to design a facial recognition system based on image processing [11]. Its core technology is facial image acquisition, image preprocessing, image feature value extraction, and image matching and recognition. Facial recognition technology is one of the most widely used technologies for image processing and analysis, which greatly facilitates people's work and life.

## 2. Facial Recognition System Design

### 2.1. Design System Architecture

**Figure 2:**
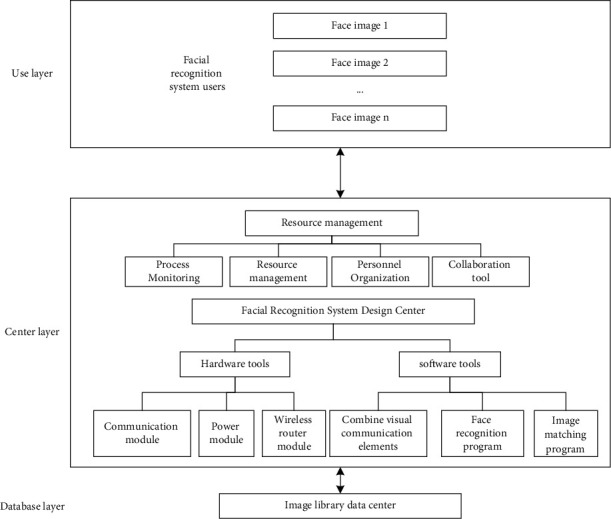
The architecture of the facial recognition system.

Based on the effect of visual communication, facial recognition technology uses image acquisition equipment to collect human facial information and input it into a computer for program calculation, and uses computer algorithm technology to process the collected facial information and analyze and extract features, so as to perform identity recognition. In a kind of technical way, the concrete facial recognition system design structure is shown in Figure 2.

The architecture includes the use layer, the central layer, and the database layer. The use layer provides an environment with visual communication effects, and the user is using the layer operating system to meet expected needs [12]. As the control center of the facial recognition system, the central layer is the most important part of the entire system. It is composed of the resource management and facial system design center. The facial system design center is responsible for maintaining the task activity process. The hardware tool communication module, power supply module, wireless router module, and software tool combine visual communication elements, facial recognition programs, and image matching programs to form a facial recognition system design center [13–15]. Resource management is responsible for the initiation and termination of mission activities. The database layer collects facial image information through the image database data center and provides the system with relevant data required for facial recognition. The user layer provides a visual communication effect environment and sends instructions to the system according to the user's needs. The central layer recognizes the facial image after receiving the instructions [16]. After the recognition is completed, the image information is fed back to the user layer, and the user obtains what they need. The facial recognition image is sent to the database layer for storage, and when the central layer needs any relevant data, it is retrieved through the database layer.

### 2.2. System Hardware Construction

**Figure 3:**
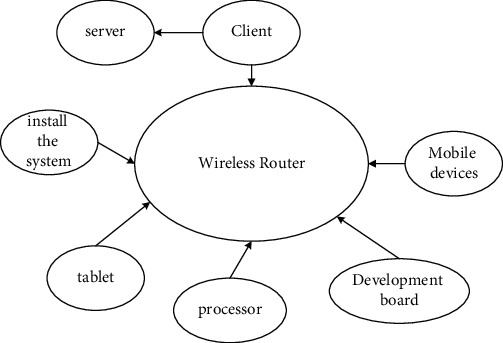
Hardware architecture of the automatic facial recognition system.

The initial task is received by the client and then assigned to the server. The client processes the facial image and submits the task to the processor. After the facial image is automatically recognized, the result of the image sequence is retrieved and used as the processor for automatic facial recognition during automatic recognition. Monitor the server for facial recognition to solve the problem of fewer network nodes. Task scheduling and facial recognition are completed by the processor and server [17]. Therefore, the most important part of the hardware of the automatic facial recognition system is to consider the memory size of the hardware. Because the smart device lacks a network port during image recognition, a wireless router needs to be used to establish a local area network (Figure 3).

### 2.3. Structural Design of the Facial System

The facial recognition program is implemented based on the OpenCV library of the Linux platform. The facial recognition program of this system is completed under the integrated development environment Qt Creator. The program uses multiple functions in OpenCV [18]. Therefore, the OpenCV library must be transplanted to the embedded system, and the program can be executed smoothly. There is a wealth of functions available in the OpenCV library. It can run on operating systems such as Linux/Window/Mac. It provides a variety of algorithms related to image processing and machine vision, and it supports multiple computer programming languages. This system mainly uses the function functions in the two header files in the OpenCV library. cv.h: this header file contains functions that can realize image processing and computer vision and other related functions, such as image processing, pattern recognition, and camera calibration. highgui.h contains the functional functions of user interaction, such as image encoding and decoding, video capture, and GUI interface. The realization of the node function is mainly divided into two parts: face detection and image matching. The following content will describe the program realization of these two parts in detail.

#### 2.3.1. Face Detection Program

Face detection is the first step of the facial recognition program, that is, the process of detecting and determining the location of the face from the image collected by the camera and separating the face from the image.


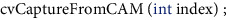


This function will open the camera after the program is executed and cyclically intercept a frame of the camera's image. The captured image is stored in the pCapture pointer of CvCapture type.


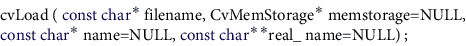


The cvLoad() function will load the file “haarcascade_frontalface_alt2.xm1” as a string. This file is an Adaboost cascaded face detection classifier based on Haar features. It is trained by extracting feature information from a large amount of face image information and is obtained. After the function loads the classifier, it will be cast to the CvHaarClassifierCascade type and assigned to the pointer cascadeo of the CvHaarClassifierCascade type.


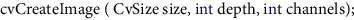


Create a single-channel image, and its return value type is IplImage type, which is the preprocessed image information to be detected, which is passed to the detection function cvHaarDetectObjects() as a parameter.


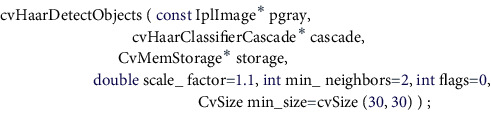


**Figure 4:**
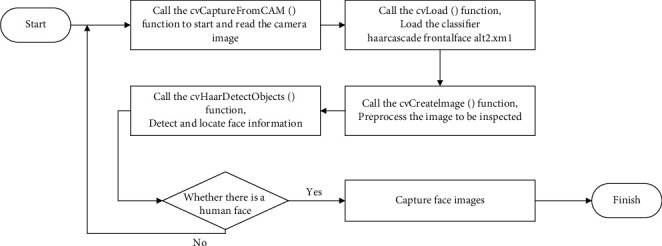
The execution flowchart of the face detection program.

The parameter pgray is the image information to be detected after preprocessing, and cascade is loaded with cvLoad( ), which is used to compare the features of the image to be detected with the Haar cascade classifier, through the cvHaarDetectObjects() function passing in the relevant parameters which realizes the function of detecting and locating the face from the image [19]. Use the above function to realize the function of the face detection part of the program, and then, save the face area image for subsequent use through the function. The realization process of the face detection function is shown in Figure 4.

#### 2.3.2. Image Matching Program

The image matching program is another important part of the realization of the function of the system recognition node. The accuracy of the recognition result directly determines the success of the function. The image matching program of this system is implemented based on the SIFT algorithm [20]. The rotation invariance and scale invariance of the SIFT algorithm can just solve various problems encountered in the actual use of the system. There are mainly the following function calls in the process of program realization.

  unread(const string& filename, int flags = 1)

You can use this function to read the image and save the image information in a Mat variable.

  class SiftFeatureDetector

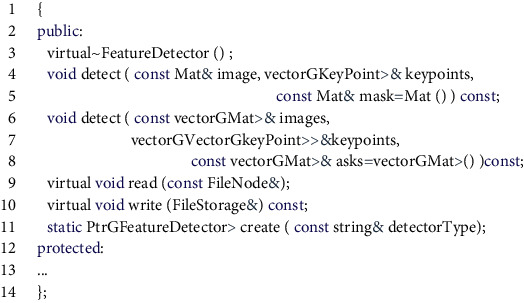
.

**Figure 5:**
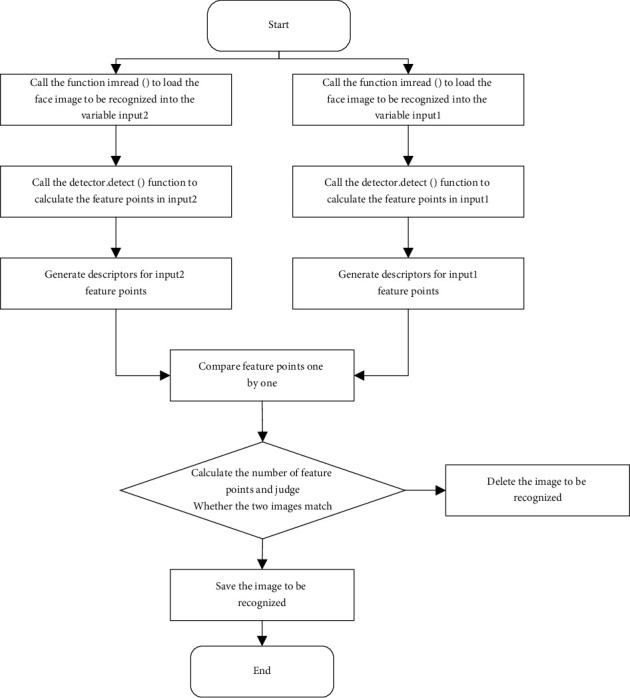
Schematic diagram of image matching program execution.

This program uses the function of detectoxdetect(). The detector is an object of the SiftFeatureDetecto: class. The function of the member function detect() it contains is to calculate the feature points in the image. The extractor.compute() function is used in the program, the extractor is an object of the SiftDescriptorExtractor class, and its member function compute() is used to specify the direction parameter for each key point and generate the key point descriptor. After the feature vectors of the two images are generated, the feature vectors of the respective key points of the two images can be used as the criterion for image similarity judgment. The process of implementing the image matching function call function is shown in Figure 5.

#### 2.3.3. Data Transmission Program Design

The master node with communication module will complete the image transmission function of the face [21]. When the face recognition node detects the current face, the facial image information of the current face will be transmitted to the designated master node through the facial recognition system In the directory, when the program detects that there is a file coming from the bus, the program will start the communication transmission module to send the image data to the facial recognition system. Among them, when the image information is transmitted through the Socket program, it is transmitted cyclically in a character array of 64 bytes. In order to make the program run normally, a communication module driver must be added to the kernel. ZTE's communication module driver is supported by files in the Linux kernel [22]. You only need to select the relevant options in the kernel compilation options. At this time, the content in the .config file needs to be changed as follows.


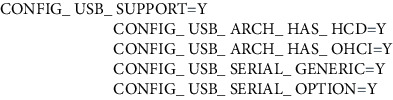


The usb_hcd structure is included in the Linux kernel, and the USB driver is described by it. The internal information, hardware resources, and he_ driver functions of the USB controller are all in this structure. The member function he_ drive in usb_ hed is a function used to operate the host controller, so it is very important. In the Linux kernel, we use the following functions to create HCD.


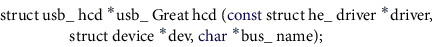


**Figure 6:**
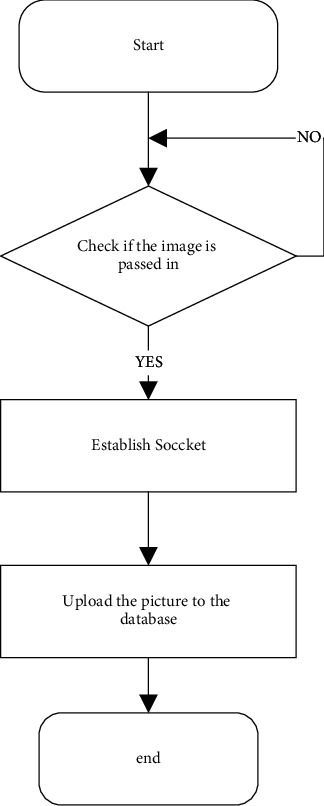
Data transmission process program diagram.

In the Kconfig file of the Linux kernel, we select “USB driver for GSM and CDMA modems.” This file contains the USB driver option configuration introduction. The location of the file is the kernel file\drivers\usb\serial directory. At this point, the driver of the communication module is added. The program running process is shown in Figure 6.

Through the above program, the current facial image information is collected and the accuracy of the recognition with the face is realized. If it is not the face recognition system, the image information will be saved and transmitted to the socket program in the communication module on the master node through the network, and the image will be transmitted to the face recognition system.

## 3. Experimental Analysis

### 3.1. Debugging of Facial Recognition Program

**Figure 7:**
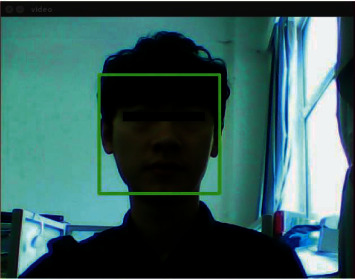
Collection result.

**Figure 8:**
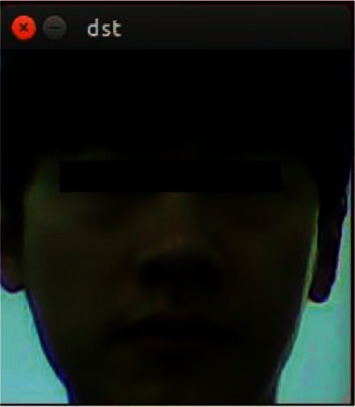
Screenshot of face.

After the initialization procedure is completed, the facial recognition system will be started by the master node. The face recognition node will assign the logical address 0 × 101, and then, the recognition program will be started to collect and recognize the image. The program needs to be compiled and debugged before being transplanted to the embedded system. When the program is executed in the embedded system, only a recognition result will be returned, and the picture collection and recognition process will not be displayed. During debugging, the display picture collection will be added for the convenience of debugging and the code of the recognition process; just comment it when the program is transplanted to the embedded system. First, after the system program is started, the camera will be called to continuously collect image information, and the collection result is shown in Figure 7:

Second, the face detection program will identify whether there is a face in the image. If there is a face, the face detection program will locate and intercept the face image. The interception result is shown in Figure 8.

Facial recognition algorithms have two important indicators: rejection rate and false recognition rate. The rejection rate is the probability that recognition is falsely rejected; that is, a copy of an image that belongs to the face database is mistakenly considered that it does not belong to the face database during recognition. The false recognition rate is the probability that a recognition error is accepted; that is, a copy of an image that does not belong to the face database is mistakenly considered to belong to the face database during recognition. In this paper, for experimental verification, the total number of samples collected was 20 people, and each person was collected 10 facial images. Due to the relatively complex external natural environment, the recognition threshold of this system is set to 0.8 after a multiperson sampling experiment, and the verification results obtained are shown in Table 1.

When the total number of collected samples is 20 people, each person has been collected 20 facial images, and after a multiperson sampling test, the recognition threshold of the system is set to 0.7, and the verification results obtained are shown in Table 2.

**Figure 9:**
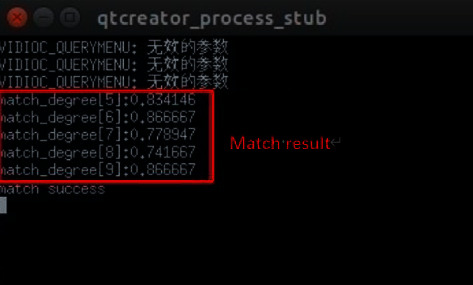
Face matching operation results.

Finally, the program compares the captured face image with the user's facial image (assuming the current tester is the user). The matching result is shown in Figure 9.

### 3.2. Data Transmission Test

When the user's facial recognition node wants to transmit image data through the asynchronous channel, the system establishes a connection to transmit the asynchronous data. The code is as follows.


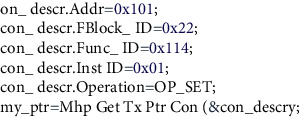


**Figure 10:**
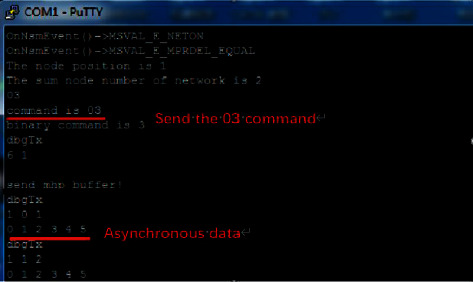
Sending asynchronous data test.

Figure 10 shows the asynchronous data sending status of the user's facial recognition node displayed in the serial port debugging tool after executing the 03 command during the asynchronous data sending test.

**Figure 11:**
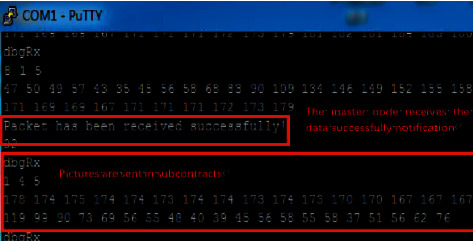
Picture transmission test.

Figure 11 is the serial port display of the face recognition node after the master node sends the command 04. When the master node sends the command 04, the facial recognition node will send the pictures collected by the camera to the master node. The image data in this system are transmitted through an asynchronous data channel and need to be divided into multiple segments because the system allocates 40 bytes of asynchronous data fields for each subsystem frame. In this way, the image information is sent to the master node in segments. Whenever an asynchronous data packet is received by the master node, the master node will return a notification message to the slave node to inform the slave node of the receiving status of the asynchronous data.

## 4. Conclusion

This paper builds a facial recognition system based on visual communication effects. From the perspective of visual communication effects, the overall design plan of the system is determined, and facial recognition technology and communication transmission technology are combined to design the facial recognition nodes and facial recognition nodes that realize the main functions of the system. The main control node with communication transmission function analyzes the existing face detection and facial recognition methods through face detection and facial recognition technology, uses the OpenCV library to design the facial recognition program, and combines the development and practical application of the system environment, the embedded environment and system test platform required for program operation are built, and the program of each node is debugged and verified. Experimental results show that the algorithm has a recognition success rate of 88.2% and a misrecognition rate of only 0.7%. Compared with traditional facial recognition technology, the success rate is significantly improved. In addition, through threshold adjustment experiments, it is shown that the optimal threshold of the algorithm is 0.8, and the recognition success rate and false recognition rate are better than other low threshold results. The feasibility of the system is verified by experiments.

## Figures and Tables

**Table 1:** Test results.

Sample	200 (%)

Recognition rate	88.2
Rejection rate	11.1
Misunderstanding rate	0.7

**Table 2:** Test results.

Sample	400 (%)

Recognition rate	85.1
Rejection rate	12.1
Misunderstanding rate	0.9

## Data Availability

The dataset can be obtained from the corresponding author upon request.
